# Marine Fish Oil Replacement with Lard or Basa Fish (*Pangasius bocourti*) Offal Oil in the Diet of Tiger Puffer (*Takifugu rubripes*): Effects on Growth Performance, Body Composition, and Flesh Quality

**DOI:** 10.3390/ani14070997

**Published:** 2024-03-24

**Authors:** Guoxu Liu, Lin Li, Shuqing Song, Qiang Ma, Yuliang Wei, Mengqing Liang, Houguo Xu

**Affiliations:** 1 State Key Laboratory of Mariculture Biobreeding and Sustainable Goods, Yellow Sea Fisheries Research Institute, Chinese Academy of Fishery Sciences, 106 Nanjing Road, Qingdao 266071, China; 2 Laboratory for Marine Fisheries Science and Food Production Processes, Laoshan Laboratory, 168 Wenhai Road, Qingdao 266237, China; 3 College of Fisheries and Life Sciences, Shanghai Ocean University, 999 Huchenghuan Road, Shanghai 201306, China

**Keywords:** fish oil replacement, by-product, alternative lipid source, flesh quality, volatile flavor compound

## Abstract

**Simple Summary:**

With the rapid development of aquaculture, searching for alternative lipid sources to fish oil has become an important task for the aqua-feed industry. Lard is regarded as a valuable alternative lipid source. Basa fish (*Pangasius bocourti*) offal oil also has a large annual production and high potential to be used in fish feeds. Evaluation of the efficacy of lard and Basa fish offal oil in fish feeds will provide useful information for their application in aquaculture. Both lard and Basa fish offal oil have high levels of saturated fatty acids and monounsaturated fatty acids. The “n-3 LC-PUFA sparing effects” of saturated fatty acids and monounsaturated fatty acids in fish feeds have been observed in many fish species. The present study was also aimed at validating the “n-3 LC-PUFA sparing effects” of saturated fatty acids and monounsaturated fatty acids in lard and Basa fish offal oil. Moreover, the effects of dietary lard and Basa fish offal oil on fish body composition and muscle quality were also evaluated.

**Abstract:**

Lard (LD) and Basa fish offal oil (BFO) have similar fatty acid profiles, both containing high contents of saturated fatty acids (SFA) and monounsaturated fatty acids (MUFA). The present study aimed to investigate the efficacy of partial or complete replacement of marine fish oil (MFO, herring oil) by LD or BFO in the diets of tiger puffer. The control diet contained 49.1% crude protein and 9.28% crude lipid content including 6% added MFO. In other diets, 1/3, 2/3, and 3/3 of the added MFO was replaced by LD or BFO, respectively. Each diet was fed to triplicate tanks of juvenile fish (initial body weight, 13.88 g). A 46-day feeding trial was conducted in a flow-through seawater system. Each diet was fed to triplicate 200-L rectangular polyethylene tanks, each of which was stocked with 30 fish. Fish were fed to satiation three times a day. The complete replacement of added MFO (replacing 65% of the total crude lipid) had no adverse effects on fish growth performance in terms of survival (>94%), weight gain (360–398%), feed intake (2.37–3.04%), feed conversion ratio (0.84–1.02), and somatic indices. The dietary LD or BFO supplementation also had marginal effects on fish body proximate composition, biochemical parameters, muscle texture, and water-holding ability, as well as the hepatic expression of lipid metabolism-related genes. Partial (2/3) replacement of added MFO by LD or BFO did not significantly reduce the muscle n-3 LC-PUFA content, indicating the n-3 LC-PUFA sparing effects of SFA and MUFA in LD and BFO. In general, dietary LD or BFO reduced the peroxidation level and led to significant changes in the muscle volatile flavor compound profile, which were probably attributed to the change in fatty acid composition. The results of this study evidenced that LD and BFO are good potential lipid sources for tiger puffer feeds.

## 1. Introduction

Marine fish oil (MFO) is rich in long-chain polyunsaturated fatty acids (LC-PUFA), such as eicosapentaenoic acid (EPA) and docosahexaenoic acid (DHA), which play important roles in fish physiology, metabolism and membrane structure and function [1,2,3,4,5]. Due to its high nutritional value, easy digestion, and efficient absorption, MFO is widely used as an ingredient for commercial aqua-feeds [6]. However, with the rapid development of aquaculture and the relative MFO shortage caused by the natural depletion of marine resources, searching for alternative lipid sources has become an urgent task for the aqua-feed industry [7,8,9]. Terrestrially sourced oils such as poultry oil, lard (LD), and beef tallow have the advantages of high yield, good availability, and relatively low price and are regarded as valuable alternative lipid sources to MFO [4]. As a by-product of pork production, LD contains abundant saturated fatty acids (SFA) and monounsaturated fatty acids (MUFA). Compared with other fatty acids, SFA and MUFA are more readily utilized for β-oxidation to generate energy [10,11,12], thus sparing the long-chain polyunsaturated fatty acids (LC-PUFA), which is called the “n-3 LC-PUFA sparing effect” [13]. Partial or complete MFO replacement with LD has proved feasible in fish diets and does not affect the growth of freshwater fish species such as largemouth bass (*Micropterus salmoides*) [14], brown trout (*Salmo trutta*) [15], gibel carp (*Carassius auratus gibelio*) [16], blunt snout bream (*Megalobrama amblycephala*) [17], and Japanese seabass (*Lateolabrax japonicus*) [18], as well as a marine species, cobia (*Rachycentron canadum*) [19]. However, complete MFO replacement with LD compromised the growth of a marine fish species, golden pompano (*Trachinotus ovatus*) [20]. This makes it necessary to carefully evaluate the efficacy of LD in the diets of specific marine fish species. In addition, alterations in the dietary fatty acid profiles caused by the addition of terrestrially sourced oils including LD may change the fish fatty acid metabolism, fillet quality, and muscle flavor [5,21,22,23,24,25]. For marine fish, in particular, the LC-PUFA content is an important quality trait of fish fillet. Therefore, how LD changes the fatty acid profile and other quality traits of marine fish also needs comprehensive studies.

As an Asian freshwater catfish, Basa fish (*Pangasius bocourti*) is widely farmed in Southeast Asia, with a total production of 2.6 million tons [26] and an export value of USD 2.26 billion in 2018 [27]. This fish species is suitable for processing into fish fillets [28]. During Basa fish processing, a large amount of waste such as head, viscera, and bone is generated, and this waste is rich in oil [29]. Basa fish offal oil (BFO) extracted from the waste, in particular the viscera, has a fatty acid composition very similar to LD [30]. BFO has not yet been used in practical aquafeeds, and its effects on growth, body composition, and fillet quality of marine fish have not been well-studied.

Tiger puffer (*Takifugu rubripes*) is an important aquaculture species in Asia, with a global production of around 20,000 tons [31,32,33]. The production of tiger puffer used to be strictly regulated by Asian governments due to the tetrodotoxin contained in this species. However, nowadays, the tetrodotoxin is no longer detectable in most farmed tiger puffer [34]. With the gradual deregulation of the marketing of this fish species, tiger puffer farming is assumed to be more prosperous in the future. Previous studies have shown that using soybean oil as the sole lipid source could reduce the growth performance of tiger puffer [35]. However, partial or complete MFO replacement with poultry oil did not affect the growth performance of tiger puffer [36]. The present study was aimed at comprehensively evaluating the efficacy of MFO replacement with LD or BFO in tiger puffer diets, in terms of growth, body composition, fatty acid composition, lipid metabolism, fillet quality, and flavor organic compound profile. This study will contribute to new lipid source screening for the feeds of tiger puffer.

## 2. Materials and Methods

### 2.1. Experimental Diets

Fishmeal, wheat meal, soybean meal, corn gluten meal, and Brewer’s yeast were used as the protein sources for the experimental diets. The fishmeal used in this study was Pollock meal (super level, steamed dried, Tecnologica De Alimentos S.A., Lima, Peru) with a protein content of 73.4% and a lipid content of 6.16% (of dry matter). The control diet contained 6% added marine fish oil (MFO, herring oil, Qingdao Surgreen Bioengineering Co. Ltd., Qingdao, China) and a total crude lipid content of 9.28%. In other diets, lard (LD) or Basa fish (*Pangasius bocourti*) offal oil (BFO) were used to replace 1/3 (33%), 2/3 (66%), and 3/3 (100%) of the added MFO, respectively. The seven isonitrogenous (approximately 48% crude protein) and isolipidic (approximately 9% crude lipid) experimental diets were designated as MFO-C (control), 1/3LD, 2/3LD, 3/3LD, 1/3BFO, 2/3BFO and 3/3BFO, respectively (Table 1). All raw ingredients for the diet preparation were sieved through an 80-mesh sieve and then evenly mixed. About 30% water was added into the dough for the pelleting. A laboratory-level pelleter was used to make the pellets with a diameter of 2 mm, and the pellets were then dried in a 55 °C-oven to a moisture content of around 8%. After the pellets were dried and cooled, they were packaged in a double-layer plastic bag and sealed. The prepared diets were stored in a cold storage room at −20 °C. The fatty acid compositions of the experimental diets and oils are presented in Table 2. The methods for the analysis of proximate composition of diets, as well as those for the analysis of dietary fatty acid composition, are available in Section 2.4.

### 2.2. Feeding Management

The feeding experiment was conducted at Yellow Sea Aquaculture Co., Ltd. (Yantai, China), with tiger puffer purchased from Hongqi Modern Fishery Industrial Park, Co., Ltd. (Rizhao, China). The fish were temporarily reared for 24 days using commercial feed to acclimate to the experimental conditions. At the beginning of the feeding experiment, the experimental fish were randomly assigned to 21 polyethylene tanks (200 L). Fish in all tanks were weighed individually, and the average initial fish weight was 13.88 g. Three replicate tanks were set up for each dietary group and 30 fish were reared in each tank. Feeding to satiation was performed three times a day (7:00, 12:00, and 18:00). Residual feeds and feces were siphoned out every day, and the tanks were regularly cleaned. The numbers of uneaten pellets, which were siphoned out, in each tank after each feeding were recorded to adjust the feed consumption data (based on an average weight of pellets). The fish were reared using flow-through seawater for 46 days. The water temperature during fish rearing was 25–28 °C, salinity 28–30, dissolved oxygen > 8 mg/L, and pH 7.6–7.9.

### 2.3. Sample Collection

At the end of the feeding experiment, the fish were fasted for 24 h before sampling and then all fish in each tank were weighed and counted. After anesthesia with eugenol (1 eugenol: 10,000 water), two fish were randomly selected from each tank for proximate composition. The body length, body weight, and visceral and liver weight were recorded for these two fish to calculate the hepatosomatic index (HSI), viserasomatic index (VSI), and condition factor (K). In addition, four fish were randomly selected from each tank for tissues sampling. The blood was collected from the tail vein of the fish using a 1 mL syringe and a 1.5 mL centrifuge tube. Heparin sodium was used to rinse the syringe to avoid coagulation during blood collection (but heparin sodium was not added into the centrifuge tube). The samples were kept at room temperature for 2 h, followed by 4 h at 4 °C, and were then centrifuged (4000× *g*, 10 min, 4 °C) to separate the supernatant. After the blood was collected, fish were dissected and the muscle and liver were subsequently collected. All tissue samples were placed in liquid nitrogen immediately for snap freezing after collection, brought back to the laboratory and stored at −80 °C. Fresh muscle samples were collected from three fish per tank for the analysis of texture and water-holding capacity. All fish handling processes, including the sampling protocols, in this study were reviewed and approved by the Animal Care and Use Committee of Yellow Sea Fisheries Research Institute. Anesthetization was conducted during fish distribution, weight measurement, and sampling to minimize the fish suffering.

### 2.4. Analysis of the Proximate Composition and Fatty Acid Profile of Fish and Diets

The proximate composition of the experimental diets and fish was analyzed according to the standard methods of the Association of Official Analytical Chemists (AOAC) [37]. The moisture, crude protein, crude lipid, and ash were assayed by the drying in 105 °C-oven to constant weight, the Kjeldahl method (FOSS KJELTEC 2300, Hillerod, Denmark), the Soxhlet extraction method (FOSS Soxtec 2050, Hillerod, Denmark), and incineration in 550 °C-muffle furnace, respectively. The fatty acid composition of oils, diets, liver, and muscle was analyzed using a gas chromatograph (GC-2010 pro, Shimadzu, Tokyo, Japan). Firstly, the chloroform methanol method was used to extract the lipids from the liver and muscle samples. Then, the saponification and esterification of the extracted oils were conducted with the following solutions in turn in a 75 °C water bath: KOH-methanol (1N) and BF3-methanol (14%). Hexane was used to extract the fatty acid methyl esters. The gas chromatograph was equipped with a flame ion detector and silica capillary column (100 m × 0.25 mm × 0.20 µm, SH-RT-2560). The column was heated firstly from 100 to 190 °C (10 °C/min); then from 190 to 200 °C (0.3 °C/min); and finally from 200 to 230 °C (4 °C/min). The temperature of the injector and detector temperature were set to be 230 °C. The temperature of the column, injector, and flame-ionization detector was 100, 250, and 300 °C, respectively. The carrier gas helium was provided at 3 mL/min. The injection volume into the gas chromatograph was 1 μL. The split ratio was set to be 50%. Standard fatty acids (a mixture of 37 fatty acids, Solarbio, Beijing, China) were used for the identification of the fatty acid peaks. The fatty acid concentration was expressed as % peak areas (% total fatty acid (TFA)).

### 2.5. Biochemical Parameters

A 10% homogenate was prepared based on 0.05 g and 0.1g muscle for the analysis of muscle malondialdehyde (MDA) and protein carbonyl, respectively. The muscle MDA and protein carbonyl, as well as the contents of total bile acid (TBA), total cholesterol (TC), triglycerides (TG), high-density lipoprotein cholesterol (HDL-C), low-density lipoprotein cholesterol (LDL-C), malondialdehyde (MDA), and protein carbonyl in serum were all assayed with commercial kits (Nanjing Jiancheng Bioengineering Institute, Nanjing, China).

### 2.6. The Quantitative Real-Time Polymerase Chain Reaction (RT-qPCR)

The total RNA was extracted from liver samples using RNAiso Plus (Accurate Biotechnology (Hunan) Co., Ltd., Changsha, China). Agarose gel electrophoresis was used to detect RNA contamination and degradation. The RNA concentration and purity were detected using a Colibri ultramicro spectrophotometer (Titerek Berthold, Bad Wildbad, Germany). Reverse transcription was performed using the Evo M-MLV RT Mix Kit with gDNA Clean for qPCR (Accurate Biotechnology (Hunan) Co., Ltd., Changsha, China). Primers for target genes and reference genes were designed based on the sequence in NCBI (Table 3) and synthesized by Qingke Biotechnology Co., Ltd. (Qingdao, China). The amplification efficiency of all primers was around 95~105%, and the linear regression coefficient (R^2^) was greater than 0.99. Adopt SYBR Green Premix Pro Taq HS qPCR Kit II (Accurate Biotechnology (Hunan) Co., Ltd., Changsha, China) and real-time fluorescence quantitative PCR instrument (Roche, Switzerland) were used for the qPCR reactions. The reaction system was a mixture of the following substances: cDNA template (1 μL), SYBR Green Pro Taq HS Premix II (2×, 5 μL), primer (forward and reverse, 10 μM, 0.3 μL for each), and sterilized water (3.4 μL). The PCR process was controlled following the program: firstly 95 °C for 30 s, and then 40 cycles of 95 °C for 5 s—57 °C for 30 s—72 °C for 30 s. At the end, a melting curve (95 °C for 10 s—65 °C for 60 s—97 °C for 1s) was drawn to confirm the product specificity. The mRNA abundance was expressed according to the 2^−ΔΔCT^ method [38].

### 2.7. Muscle Texture and Water-Holding Capacity

After sampling, three fresh, smooth, and intact muscle samples were selected from each tank for the flesh quality assay. The hardness, adhesiveness, cohesion, elasticity, stickiness, and chewiness of each flesh sample were measured with a texture analyzer (TMS-PRO, Food Technology Corporation, Sterling, VA, USA) equipped with a 25 N gravity sensor. The measurement conditions for the texture analyzer were as follows: probe diameter, 8 mm; test speed, 30 mm/min; and deformation ratio, 30%. The muscle samples used for texture analysis were sub-divided for the subsequent analysis of centrifugal loss and steaming loss, according to the requirements of the following methods. 

For the analysis of centrifugal loss, a 2 g muscle sample (W1) was weighed and placed in a 10 mL centrifuge tube with absorbent paper at the bottom. The sample was centrifuged at 1000× *g* for 10 min. After the supernatant was removed, the surface moisture was wiped off, and the muscle sample with absorbent paper was weighed again (W2). For the analysis of steaming loss, a 3 g muscle sample (W1) was weighed and wrapped with gauze to prevent looseness. The sample was then steamed in a steamer for 5 min. After the removal of supernatant, the surface moisture of the muscle sample was wiped off, and the muscle sample was cooled and weighed (W2). The steaming (centrifugal) water loss rate (%) = 100 × (W1 − W2)/W1.

### 2.8. Analysis of Volatile Organic Compounds in the Muscle

Gas chromatography−ion migration spectrometry (GC-IMS) was used to determine the volatile organic compound composition in the muscle. Before analysis, muscle samples (5 g) were placed in headspace injection bottles (20 mL) and incubated (60 °C for 15 min). A total of 500 μL gas was injected into the machine. The injection needle temperature was 85 °C. The carrier gas was high-purity nitrogen (99.999%), and an automatic sampler was used for non-split injection. The chromatographic column type was MXT-5 (RESTEK, Bellefonte, PA, USA; 15 m × 0.53 mm × 1.0 μm). The column and IMS temperature was 60 and 45 °C, respectively.

### 2.9. Statistical Methods

Calculations are according to the following equations:

Weight gain (WG, %) = (FBW − IBW) / IBW × 100;

Feed intake (FI, %) = feed dry weight / [experimental days × (IBW + FBW) / 2] × 100;

Feed conversion ratio (FCR) = feed intake / weight gain;

Survival (%) = final fish number / initial fish number × 100;

Hepatosomatic index (HSI, %) = (liver weight / body weight) × 100;

Viserasomatic index (VSI, %) = (viscera weight / body weight) × 100;

Condition factor (K, g/cm^3^) = weight of fish / length of fish^3^ × 100;

where IBW and FBW represent initial and final body weight, respectively. 

All experimental data were analyzed using SPSS 16.0 for one-way ANOVA and Tukey’s test. Data from each tank were regarded as a replicate in the statistics. When *p <* 0.05, there was a significant difference. The results are expressed as the mean ± standard error. 

## 3. Results

### 3.1. Growth Performance, Somatic Indices, and Body Composition

The substitution of LD or BFO for MFO did not significantly (*p >* 0.05) affect all the growth parameters of tiger puffer, including FBW, WG, FCR, and FI (Table 4). The survival of all groups was higher than 94%, and no significant difference was observed in survival among groups. There was no significant difference in somatic indices such as K, HIS, and VSI (Table 4). 

The replacement of MFO with LD or BFO had no significant effect on the proximate composition of whole fish body, muscle, and liver (Table 5), except that the crude lipid of the muscle in the 2/3LD and 1/3BFO groups were significantly lower compared to the other groups (*p <* 0.05).

### 3.2. Fatty Acid Compositions in Muscle and Liver

In the muscle, high levels of MFO replacement by LD (1/3LD and 3/3LD) or BFO (2/3BFO and 3/3BFO) significantly (*p <* 0.05) increased the 18:1n-9 content (Table 6). The increase in LD and BFO levels linearly increased the 18:2n-6 content but linearly decreased the 18:3n-3 content. Compared to the MFO-C (control) group, only the 3/3BFO group significantly (*p <* 0.05) decreased the 20:5n-3 (EPA) and 22:6n-3 (DHA) contents. The MFO replacement by LD or BFO did not significantly (*p >* 0.05) change the saturated fatty acid (SFA) contents. 

In the liver, the MFO replacement by LD or BFO did not significantly (*p >* 0.05) change the SFA contents either (Table 7). For the MUFA, the increase in LD and BFO levels linearly decreased the contents of 16:1n-7 and 20:1n-9 but linearly increased the 18:1n-9 content. For the n-6 and n-3 fatty acids, similar to the muscle, the supplementation of LD or BFO increased the 18:2n-6 content but decreased the contents of 20:4n-6 (ARA), 18:3n-3, EPA, 22:5n-3, and DHA. However, the decrease in n-3 LC-PUFA in the liver was more drastic compared to that in muscle. The 2/3LD and 2/3BFO groups already significantly (*p <* 0.05) decreased the EPA and DHA contents in the liver, although for 22:5n-3, only 3/3LD and 3/3BFO significantly (*p <* 0.05) decreased its content.

### 3.3. Serum and Muscle Biochemical Parameters

In general, the dietary supplementation of LD or BFO had marginal effects on the serum biochemical indicators of tiger puffer such as TBA, TG, TC, HDL-C, and LDL-C (Table 8). However, in both muscle and serum, the MFO replacement by LD or BFO tended to reduce the contents of MDA and protein carbonyl.

### 3.4. Hepatic mRNA Expression of Lipid Metabolism-Genes

The replacement of MFO with LD or BFO had marginal effects on the expression of lipid metabolism-related genes in the liver of the tiger puffer (Table 9). The dietary LD supplementation generally decreased the gene expression of *cyp7a1*, and the 1/3LD group had a significantly (*p <* 0.05) lower expression compared to the MFO-C group.

### 3.5. Muscle Texture and Water-Holding Capacity

The replacement of MFO with LD or BFO did not significantly (*p >* 0.05) affect the muscle texture and water-holding capacity of tiger puffer (*p >* 0.05) (Table 10).

### 3.6. Analysis of Volatile Organic Compounds in the Muscle

**Figure 1:**
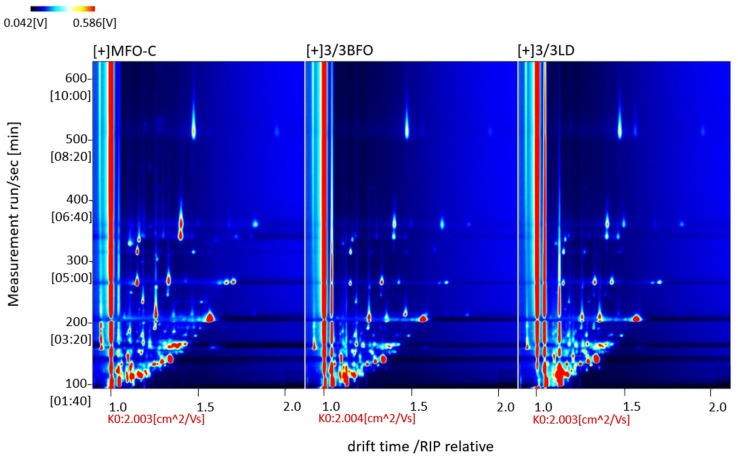
Spectra of volatile flavor components in the samples (top view). The vertical axis represents the retention time (s) of gas chromatography, and the horizontal axis represents the ion migration time (normalization treatment). The red vertical line at abscissa 1.0 represents the normalized reaction ion peak (RIP) peak and each point around RIP represents a volatile organic compound. The substance abundance is indicated by the color brightness. The white color shows a lower substance concentration, while the red color shows a higher concentration.

**Figure 2:**
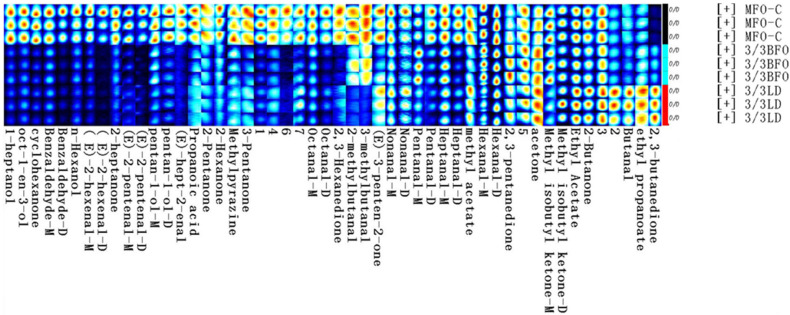
Gallery plot of the muscle volatile flavor compounds. All selected signal peaks in a sample were aligned in a row. The signal peaks of the same volatile organic compound in different samples were aligned in a column. A brighter color represents a higher concentration of a substance. The numbers represent unidentified substances in the migration spectrum library.

**Figure 3:**
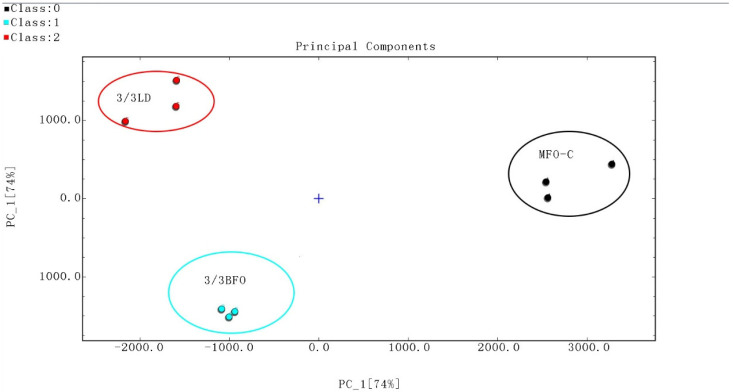
Principal component analysis (PCA) of volatile flavor compounds in the muscle. A larger distance between samples represents a more significant difference.

**Figure 4:**
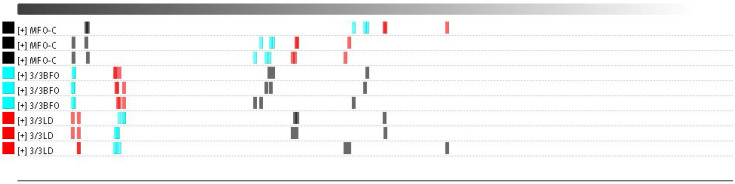
Euclidean distance of volatile flavor compounds in the muscle. A larger distance between samples represents a more significant difference.

A total of 50 volatile flavor components were detected from all muscle samples, of which 43 were successfully identified (Table 11, Figure 1 and Figure 2). Most of these compounds were small-molecular alcohols, ketones, and aldehydes. Aldehydes are the most abundant volatile organic compounds in the muscle of tiger puffer, followed by ketones. The difference in volatile organic compounds between MFO-C and 3/3LD was greater than that between MFO-C and 3/3BFO (Figure 3 and Figure 4). Compared with the MFO-C group, the 3/3LD group showed a lower abundance of octanal-M, octanal-D, 1-heptanol, oct-1-en-3-ol, cyclohexanone, benzaldehyde-M, benzaldehyde-D, n-hexanol, 2-methylbutanal, (E)-2-hexenal-M, (E)-2-hexenal-D, 2-heptanone, 2-pentanone, 2,3-hexanedione, (E)-2-pentenal-M, (E)-2-pentenal-D, 3-methylbutanal, pentan-1-ol-M, pentan-1-ol-D, 3-pentanone, 2-hexanone, (E)-hept-2-enal, methylpyrazine, propanoic acid, heptanal-M, pentanal-D, heptanal-D, and methyl isobutyl ketone-M, but a higher abundance of 2,3-butanedione, ethyl propionate, butyraldehyde, acetone, 2,3-pentanedione, and 2-butanone (Figure 2). Compared with the MFO-C group, the 3/3BFO group showed a lower abundance of 3-pentanone, (E)-2-pentenal-M, (E)-2-pentenal-D, 2,3-hexanedione, (E)-2-hexenal-M, (E)-2-hexenal-D, (E)-hept-2-enal, benzaldehyde-M, benzaldehyde-D, octanal-M, octanal-D, 1-heptanol, oct-1-en-3-ol, cyclohexanone, n-hexanol, 2-heptanone, pentan-1-ol-M, pentan-1-ol-D, 2-pentanone, and heptanal-D, but a higher abundance of 2,3-butanedione, ethyl propionate, 2,3-pentanedione, and acetone (Figure 2).

## 4. Discussion

Although Basa fish offal oil (BFO) is derived from fish, it contains little LC-PUFA. This is due to the fact that LC-PUFA tend to be selectively retained in the brain, eye, and muscle of fish [10,11,39]. In terms of fatty acid profile, BFO is very similar to lard (LD) [30], which is why the efficacy of these two oils in fish diets was compared in the present study. The results of this study showed that neither oil significantly compromised the growth of juvenile tiger puffer, regardless of replacement level. This indicates the high potential of LD and BFO as a lipid source for tiger puffer feeds. The high efficiency of LD in feeds has also been observed in other species. It has been observed that LD can replace as high as 100% MFO in the diets of freshwater species such as brown trout [15], surubim (*Pseudoplatystoma coruscans*) [40], grass carp (*Ctenopharyngodon idella*) [41], rice field eel (*Monopterus albus*) [42], and Amur sturgeon (*Acipenser schrenckii*) [43]. In marine fish species such as cobia [19], LD was found to be able to replace 50% MFO in the diets. However, for hybrid tilapia (*Oreochromis niloticus × O. aureus*), complete MFO replacement by LD significantly reduced the fish growth [44]. Also, complete MFO replacement with LD compromised the growth of a marine fish species, golden pompano [20]. These results indicate that the efficacy of LD in fish diet could be species-specific. The LD contains high levels of SFA and MUFA, which could be less digestible in some species such as tilapia and golden pompano. 

Different from LD, studies on the use of BFO in fish feeds have not been available. Relevant studies are needed because BFO has been used in practical fish feeds. Also, it is notable that although statistically the use of LD and BFO did not compromise the fish growth, numerically, complete MFO replacement with LD and BFO lowered the growth of tiger puffer. It was speculated that longer-term feeding durations may enlarge this growth reduction. In addition, if LD and BFO were compared at the same replacement level, the BFO groups had lower growth numerically. The LD and BFO had very similar fatty acid compositions, except that the 18:0 content in LD was much higher than in BFO (16% vs. 7.83%). Our previous studies have evidenced that tiger puffer has high 18:0 contents in the muscle and has a high capacity to utilize SFA [45,46]. This could explain the better growth of tiger puffer fed LD compared to those fed BFO. Another reason which could explain this difference could be the different lipid structures between LD and BFO. Further studies are needed in this area. 

Dietary LD and BFO affected the lipid content in the muscle of tiger puffer. Partial replacement of MFO with LD (2/3LD) or BFO (1/3BFO) significantly decreased the crude lipid content in the muscle. This was different from previous results. In general, complete or high levels (over 75%) of MFO replacement can easily lead to an increase in lipid content [5]. In rainbow trout (*Oncorhynchus mykiss*) [47], grass carp [41], and brown trout [15], the replacement of MFO with LD did not affect muscle lipid content, but there was an increasing trend with the increase in MFO replacement levels. 

In general, the fatty acid composition of tiger puffer tissues reflects those of the diets, which was consistent with the previous findings [16,18,43]. However, the hypothesis of this study, namely, the “n-3 LC-PUFA sparing effect” of SFA and MUFA in LD and BFO, was still validated to some extent. Statistically, only complete MFO replacement with LD or BFO significantly reduced the muscle EPA and DHA content, suggesting that a high level (2/3) of MFO replacement with LD or BFO led to an acceptable change in muscle LC-PUFA content. The DHA content in the diets 3/3LD and 3/3BFO was 24.9% and 24.7% that of the diet MFO-C, respectively (the ratio for EPA was 42.5% and 43.6%, respectively). However, the muscle DHA content in the groups 3/3LD and 3/3BFO was 72.5% and 61.5% that of the MFO-C group, respectively (the ratio for EPA was 78.0% and 65.8%, respectively). It was apparent that DHA and EPA were selectively deposited in fish muscle. In contrast, opposite trends were observed for SFA and MUFA, which were selectively utilized. For 16:0, in spite of the increase in content in the diet with increasing LD or BFO levels, most MFO replacement groups showed a decreased 16:0 content in the muscle compared to MFO-C. These results indicate that the MUFA and SFA, in particular 16:0, were readily utilized by tiger puffer to spare the n-3 LC-PUFA, which have been widely demonstrated in other species [13,19,48]. The 1/3BFO group even had higher levels of muscle DHA and EPA than the MFO-C group, although there were no significant differences.

If the muscle fatty acid-regulating effects were compared between LD and BFO, no significant difference was observed. As mentioned previously, the two lipid sources, LD and BFO, had very similar fatty acid profiles, except that LD had a higher 18:0 content (16.0% vs. 7.83%) but a slightly lower 16:0 (25.5% vs. 29.4%) content than BFO. The 18:0 has been known as a less important fatty acid, which is a poor substrate for triacylglycerol synthesis and β-oxidation and has a relatively lower apparent digestibility coefficient in fish [10,49]. This could explain the lower influence of the 18:0 difference on the muscle fatty acid composition of tiger puffer. However, a recent meta-analysis showed the accumulation of 18:0 in some functional tissue of fish such as brain, heart, and eye, indicating the potential function of 18:0 in these tissues [5]. The functions of 18:0 in fish is worthy of further investigation. 

For tiger puffer, the liver is also an edible part. In general, the response of liver fatty acids to dietary changes was similar to the muscle, but the change in liver fatty acid compositions, in particular LC-PUFA, by diets was more drastic compared to muscle. Only low levels of MFO replacement (1/3LD and 1/3BFO) maintained the liver DHA and EPA contents to a level comparable to the MFO-C group. The LC-PUFA tend to be selectively deposited in polar lipids [11]. As a lean species, tiger puffer has high polar lipid contents in the muscle [50]. These factors could explain the more drastic changes in liver LC-PUFA in response to dietary LD or BFO supplementation. However, there was also no significant difference in liver 16:0 content among groups in spite of the variation in 16:0 content among the diets. To some extent, this also validated the LC-PUFA sparing effects of SFA in LD and BFO. 

The replacement of MFO with LD and BFO had little effect on the biochemical indices in the muscle and serum of tiger puffer. However, the LD or BFO tended to reduce the contents of protein carbonyl and MDA, which is the peroxidation product of protein and lipid, respectively. The decrease in LC-PUFA, which are relatively easily peroxidized, could be the main reason for decreased peroxidation, as observed in studies on Japanese seabass [51], large yellow croaker (*Larimichthys crocea*) [52], and black seabream (*Acanthopagrus schlegelii*) [53], as well as a previous study on tiger puffer [36].

The gene expression results indicate that both LD and BFO have marginal effects on the expression of lipid metabolism genes in tiger puffer. Dietary supplementation of LD significantly down-regulated the expression of *cyp7a1* in the liver. This was not consistent with a similar study on tiger puffer, which showed that MFO replacement with poultry oil up-regulated the *cyp7a1* gene expression in the liver [36]. *Cyp7al* is a key regulatory enzyme for bile acid biosynthesis using cholesterol as a substrate [54]. The relative abundance of cholesterol and bile acids in alternative oils may largely influence the gene expression of *cyp7a1*. 

Previous studies have shown that dietary oil sources could affect the quality of fish fillets [55,56]. For example, replacing MFO with vegetable oil can slightly reduce the fillet hardness of seabream (*Sparus aurata*), which may be related to the high lipid content in the fish flesh [57]. However, in this study, the MFO replacement by LD or BFO did not change the muscle texture and water-holding capacity, similar to what was observed in gibel carp [16]. The discrepancy may be mainly related to muscle lipid content and fish size [16]. 

The profile of volatile flavor compounds (VFCs) in fish flesh largely contributes to the flesh flavor [58]. The VFCs in the muscle of puffer fish mainly consist of aldehydes, ketones, alcohols, phenols, and heterocyclic compounds containing nitrogen and sulfur [59]. The complete MFO replacement with LD or BFO significantly affected the VFC profile in the muscle. Moreover, more similar VFC profiles were observed between the two alternative oil groups than between alternative oil groups and MFO-C groups. This indicates that the fatty acid profile may be a primary factor determining the muscle VFC profile. Many VFCs are metabolites of fatty acids and can be readily influenced by dietary lipid sources [47,60,61,62]. Compared to the 3/3BFO or 3/3LD groups, the muscle of the MFO-C group was richer in aldehydes such as benzaldehyde, 2-hexenal, 2-pentenal, pentenal, octanal, and heptanal, alcohols such as 1-heptanol, oct-1-en-3-o1, n-hexanol, and pentan-1-ol, as well as ketones such as cyclohexanone, 2-heptanone, 2-pentanone, 2-hexanone, 3-pentanone, and 2,3-hexanedione. Heptanal, which has a fresh and nutty flavor [63], is formed by the oxidation of n-9 MUFA and n-6 PUFA [64]. Oct-1-en-3-o1, which has a fishy and fatty flavor [63,65,66], is formed from the oxidation of arachidonic acid by 12-lipoxygenase [64]. Most of the aldehydes and ketones mentioned above are also derived from lipid oxidation. (E)-2-hexenal and (E)-2-pentenal, which have a pleasant green or nutty aroma, are identified from the action of 15-lipoxygenase on linolenic acid and DHA [64,67], but octanal, which is also derived from either oleic acid or linoleic acid oxidation [61], has grassy [65,66,68] and leafy [65] flavors. A ketone product of lipid oxidation, cyclohexanone, has an earthy flavor [64], and another ketone product of linoleic acid oxidation [69], 2-pentanone, has blue cheese and fruity flavors [70]. 

Although major differences in VFC profile were observed between alternative oil groups and the MFO-C group, minor differences were also found between the two alternative oil groups. The 3/3BFO group was rich in 2-methylbutanal and 3-methylbutanal, which are generated from leucine and isoleucine, respectively, via Strecker degradation, and have a strong burnt and apple odor flavor, respectively [71]. The 3/3LD group was rich in (E)-3-penten-2-one, 2,3-butanedione, ethyl propanoate, and butanal. The 2,3-butanedione, which has a buttery and caramel odor [72], can be formed by the Maillard reaction [73], while butanal, which has a pungent and intense flavor, might be attributed to the synergistic effect of endogenous enzymes and microorganisms in the storage process [73]. Therefore, the characteristic flavors of the 3/3BFO and 3/3LD groups could be mainly related to the production processes and storage condition of these two oils. Moreover, the distance between 3/3LD and MFO-C was larger than that between 3/3BFO and MFO-C, which could also be explained by the oil production processes and storage condition. 

## 5. Conclusions

In the diet of juvenile tiger puffer, lard (LD) or Basa fish offal oil (BFO) can completely replace the added marine fish oil (MFO) (replacing 65% of the total crude lipid), without adverse effects on fish growth. Partial MFO replacement (2/3 of added MFO) by LD or BFO did not significantly reduce the muscle n-3 LC-PUFA content. The n-3 LC-PUFA sparing effects of SFA and MUFA in LD and BFO were validated to some extent. Dietary LD or BFO supplementation had marginal effects on fish body proximate composition, biochemical parameters, muscle texture, and water-holding ability, as well as the hepatic expression of lipid metabolism-related genes. However, dietary LD or BFO leads to significant changes in the muscle volatile flavor compound profile, probably related to the change in fatty acid composition. The results of this study evidenced that LD and BFO are good potential lipids sources for tiger puffer feeds.

## Figures and Tables

**Table 1:** Formulation and proximate composition of the experimental diets (% dry matter basis).

Ingredients	MFO-C	Lard (LD)			Basa Fish Offal Oil (BFO)		
		1/3LD	2/3LD	3/3LD	1/3BFO	2/3BFO	3/3BFO
Fish meal ^1^	42.0	42.0	42.0	42.0	42.0	42.0	42.0
Wheat meal	20.7	20.7	20.7	20.7	20.7	20.7	20.7
Soybean meal	14.0	14.0	14.0	14.0	14.0	14.0	14.0
Corn gluten meal	8.00	8.00	8.00	8.00	8.00	8.00	8.00
Brewer’s yeast	5.00	5.00	5.00	5.00	5.00	5.00	5.00
Mineral premix ^2^	0.50	0.50	0.50	0.50	0.50	0.50	0.50
Vitamin premix ^2^	1.00	1.00	1.00	1.00	1.00	1.00	1.00
Monocalcium phosphate	1.00	1.00	1.00	1.00	1.00	1.00	1.00
L-ascorbyl-2-polyphosphate	0.20	0.20	0.20	0.20	0.20	0.20	0.20
Choline chloride	0.20	0.20	0.20	0.20	0.20	0.20	0.20
Betaine	0.30	0.30	0.30	0.30	0.30	0.30	0.30
Ethoxyquin	0.02	0.02	0.02	0.02	0.02	0.02	0.02
Calcium propionate	0.10	0.10	0.10	0.10	0.10	0.10	0.10
Soya lecithin	1.00	1.00	1.00	1.00	1.00	1.00	1.00
Marine fish oil ^3^	6.00	4.00	2.00	0.00	4.00	2.00	0.00
Lard	0.00	2.00	4.00	6.00	0.00	0.00	0.00
Basa fish offal oil	0.00	0.00	0.00	0.00	2.00	4.00	6.00
Proximate composition							
Crude protein	49.1	48.0	47.9	48.4	47.9	47.9	48.2
Crude lipid	9.28	9.48	9.21	9.20	9.17	8.66	9.50
Moisture	5.06	4.55	4.30	5.72	3.52	3.63	4.19
Ash	9.96	9.97	9.97	9.73	10.1	10.1	9.58

^1^ The fishmeal used in this study was Pollock meal (super level, steamed dried, Tecnologica De Alimentos S.A., Lima, Peru) with a protein content of 73.4% and a lipid content of 6.16% (of dry matter). ^2^ Vitamin premix and mineral premix, specially designed for marine carnivorous fish, were provided by Qingdao Master Biotech Co., Ltd. (Qingdao, China). ^3^ The added fish oil used in this study was herring oil, which was purchased from Qingdao Surgreen Bioengineering Co. Ltd., Qingdao, China.

**Table 2:** Fatty acid composition of the experimental diets and oils (%TFA).

Fatty Acid	Diet							Oil		
	MFO-C	Lard (LD)			Basa Fish Offal Oil (BFO)			MFO	LD	BFO
		1/3LD	2/3LD	3/3LD	1/3BFO	2/3BFO	3/3BFO			
12:0	0.06	0.08	0.07	0.07	0.07	0.07	0.07	0.05	0.07	0.09
14:0	4.24	3.74	2.96	2.29	4.21	3.69	3.67	4.38	1.31	3.62
16:0	20.6	22.5	23.4	25.3	24.5	26.8	30.3	15.6	25.5	29.4
18:0	5.19	7.34	9.60	12.0	6.20	7.21	8.14	3.74	16.0	7.83
SFA	31.0	34.5	36.6	40.2	35.7	38.3	42.7	25.1	43.4	41.2
16:1n-7	4.79	4.14	3.41	1.42	4.01	3.47	2.22	0.08	2.02	0.98
18:1n-9	15.0	19.0	23.3	28.2	19.1	23.1	26.3	12.6	37.6	39.4
20:1n-9	1.99	1.68	1.41	1.22	1.96	1.49	1.28	1.17	0.43	0.60
22:1n-9	0.21	0.25	0.11	0.07	0.19	0.12	0.07	0.21	0.06	0.05
24:1n-9	0.39	0.12	0.21	0.13	0.36	0.40	0.09	0.08	0.01	0.01
MUFA	27.9	30.0	33.1	35.5	30.1	32.4	33.1	18.6	43.3	41.3
18:2n-6	12.7	13.7	14.8	16.3	12.9	14.1	15.2	11.4	10.4	10.2
18:3n-6	0.65	0.42	0.22	0.03	0.48	0.29	0.08	0.92	0.03	0.09
20:3n-6	3.14	2.05	1.05	0.06	2.47	1.05	0.11	4.52	0.01	0.06
20:4n-6	0.12	ND	ND	ND	ND	ND	ND	0.12	ND	0.01
n-6PUFA	17.0	16.8	16.8	16.6	16.4	15.7	15.8	17.9	10.9	10.9
18:3n-3	0.05	0.04	0.02	0.47	0.07	0.04	0.02	0.10	0.02	0.02
20:5n-3	7.93	6.31	4.85	3.37	6.13	4.83	3.46	6.28	0.13	0.00
22:5n-3	1.62	0.63	0.06	0.02	0.06	0.04	1.09	1.30	0.04	0.01
22:6n-3	13.3	10.9	7.77	3.31	10.7	7.77	3.28	11.9	0.01	0.01
n-3PUFA	24.1	18.8	13.6	7.77	17.8	13.6	8.37	20.5	0.20	0.52
n-3/n-6	1.42	1.12	0.81	0.47	1.09	0.87	0.53	1.15	0.02	0.05

In addition to the fatty acids listed in the table, the total fatty acids include 13:0, 15:0, 17:0, 20:0, 21:0, 22:0, 14:1n-5, 15:1n-5, 17:1n-7, 20:2n-6, 22:2n-6, and 20:3n-3. ND: non-detectable.

**Table 3:** Sequences of the primers used.

Primer	Sequence (5′-3′)	GenBank Reference	PL (bp)
Lipogenesis			
*fas*-F	CTTTGCCGCTGTCATTCG	XM_011619859.1	78
*fas*-R	TGTCTCAACCCATTTGTAGTCG		
β-oxidation			
*cpt-1*-F	GGGGTTTGTGGTCAAGTTAGG	XM_011607269.1	186
*cpt-1*-R	ATAGATCCGTGGCGCTCAT		
*acox1-q*F	GCACGGCATCGCAAGTAAC	XM_029850253.1	145
*acox1-q*R	GAGATCGAAGGCATCCACC		
Biosynthesis of glycerides			
*gpat*-F	CCCGTTCACAAATCCCACA	XM_011621885.1	235
*gpat*-R	GGCACAACAACTCCTCCGTAT		
*dgat1*-F	TGGTTTGTGAGCCGTTTCC	XM_003969352.2	185
*dgat1*-R	CTGGCATTCGTTTGACTTCG		
*mgat2a*-F	AAAGGCTTCATTAAATTGGC	XM_003978609.3	223
*mgat2a*-R	TGATGGCTTGTCTGTAGGG		
Hydrolysis of glycerides			
*atgl*-F	CCAACCTCTACAGGGTCTCA	XM_003967696.3	119
*atgl*-R	GTTTAGCAGCCCGTTCTTC		
*daglα*-F	CTGTTGGTGGAGTTGGTGTATG	XM_011610175.1	72
*daglα*-R	ATCAGAGCACGGCTGGTAAT		
*hsl*-F	CTCTTGCTATCGGTCTTGTGG	XM_011621066.1	113
*hsl*-R	TTCTGGGTCAATGGCATACTT		
*mgll*-F	CCATCCAGTCAAAGTGGGTCT	XM_003963030.2	110
*mgll*-R	CATCAGCTGCATGCCGAA		
Lipid digestion			
*bsal*-F	TTGAAGATGACTGACCCCGA	XM_003978375.2	162
*bsal*-R	GATGTCTGCTGCGTTGTGAA		
*lp*-F	CGTTTTCTCCTGTTCACCC	XM_029832009.1	97
*lp*-R	GACTCGTCCTCATCCCACT		
Lipid transport			
*lpl*-F	AGGGTCCACATCCGCAAA	NM_001305600.1	157
*lpl*-R	GTTTCTCCTTGCGGCTCAT		
*lipc*-F	GCGGCTTCAACAGCAGTAA	XM_011610357.1	215
*lipc*-R	GAGGTGCGCTATGTCTTTCC		
*fabp1*-F	CCATCGGTCTCCCTGATGAAG	XM_003974807.3	121
*fabp1*-R	TTGACCGTTACCTTCGGTCC		
*fatp1*-F	ATTGCAGACACCACAGGGAG	XM_003964742.3	219
*fatp1*-R	ATATCGTGACGCTCGTGCAT		
*apoa1-*F	CGATGACGCCGAGTACAAA	AB183289.1	104
*apoa1*-R	CGGTTATGGGAGAAACGCTA		
*apoa4*-F	TGCTTTCTGGGACTATGTTGC	NM_001078591.1	124
*apoa4*-R	GTTGACTTTGTCGGCACTCTC		
*apob100*-F	AGGGACATAGTCAAACCAAGGA	XM_011619944.1	127
*apob100*-R	AGAACACGAAGGCTGGACAC		
*apoe1*-F	TATTCAGACCCGCACCTCA	NM_001078592.1	201
*apoe1*-R	ATTTCCTCCATCTTGTCCTCC		
Lipid metabolism-related transcriptional factors			
*srebf1*-F	TTTCAGCATCCCACCTTCC	XM_011603881.1	158
*srebf1*-R	GGTGAACCGTGAGGACAACTA		
*pparα1*-F	TCAGTAGTTTATGGGTTGGTGG	NM_001097630.1	119
*pparα1*-R	GCGTGGACTCCGTAGTGGTA		
*pparα2*-F	CCAGAAGAAGAACCGCAACA	NM_001097629.1	149
*pparα2*-R	CCTCTTTCTCCACCATCTTGTT		
*pparβ*-F	AGCTGGAATACGACCGATGT	AB275887.1	249
*pparβ*-R	TCTTCAGGTAGGCGGAGTTG		
*pparγ*-F	CGCTGTCCCGACATCTGTAT	NM_001097627.1	146
*pparγ*-R	GAACTGCTCGCCTTCCATT		
*fxr*-F	GTGAACGACCACAAGTTTACCC	XM_003967283.2	166
*fxr*-R	AGACCAACAGATTACACCGGAT		
*lxrα*-F	GTGACGCACCACTAACAGCA	XM_011609917.1	191
*lxrα*-R	CTGACAACACCGAGCAAGACT		
*hnf4α*-F	GAGCCACGGGCAAACACTA	XM_011619034.1	199
*hnf4α*-R	AGGGTCCTACCTTCTTTCTTCAT		
*lrh-1*-F	CGCTGACATGCTGCCTAAA	XM_003974281.2	140
*lrh-1*-R	TCTCGTCCAAGTCTTCGTCAT		
Cholesterol and bile acid biosynthesis			
*hmgcr-*F	GCTGCTGGCAATCAAGTACAT	XM_003974466.2	237
*hmgcr*-R	AAACATACAACTCCTTCCTACAGC		
*cyp7α1*-F	CCTACCTGCTACCTTCTGGAGT	XM_003975521.2	143
*cyp7α1*-R	TCCTCTTTGGCAACACGAA		
Reference gene			
*RPL*13-F	ACTGTGACTTGTCACCTTTGT	XM_011610267.2	146
*RPL*13-R	CCTGCGGATCTTCCTAGCTG		
*EF*1*α*-F	TTGGAGGCATTGGAACTGT	NM_001037873.1	86
*EF*1*α*-R	GTTGACGGGAGCAAAGGT		

*fas*: fatty acid synthase; *cpt-1*: carnitine O-palmitoyltransferase-1; *acox1*: acylCoA oxidase 1, palmitoyl; *gpat*: glycerol-3-phosphate acyltransferase; *dgat1*: diacylglycerol O-acyltransferase 1; *mgat2a*: 2-acylglycerol O-acyltransferase 2-A-like (LOC101069338); *atgl*: adipose triglyceride lipase (patatin-like phospholipase domain containing 2 (pnpla2)); *daglα*: diacylglycerol lipase, alpha; *hsl*: hormone-sensitive lipase; *mgll*: monoglyceride lipase; *bsal*: bile acid activated lipase; *lp*: inactive pancreatic lipase-related protein 1-like (LOC101064949); *lpl*: lipoprotein lipase; *lipc*: lipase, hepatic; *fabp*: fatty acid binding protein; *fatp*: fatty acid transport protein (solute carrier family 27 member 1 (slc27a1)); *apo*: apolipoprotein; *srebf1*: sterol regulatory element binding transcription factor 1; *ppar*: peroxisome proliferator-activated receptor; *fxr*: farnesoid X receptor (nuclear receptor subfamily 1, group H, member 4, NR1H4); *lxrα*: liver X receptor alpha (nuclear receptor subfamily 1, group H, member 3, NR1H3); *hnf4α*: hepatocyte nuclear factor 4, alpha; *lrh-1*: liver receptor homolog-1 (nuclear receptor subfamily 5, group A, member 2, NR5A2); *hmgcr*: 3-hydroxy-3-methylglutaryl-CoA reductase; *cyp7a1*: cholesterol 7-alpha-hydroxylase (cytochrome P450 family 7 subfamily A member 1); PL: sequence length.

**Table 4:** Growth performance and somatic indices of experimental tiger puffer (mean ± standard error).

Parameters	MFO-C	Lard (LD)			Basa Fish Offal Oil (BFO)			*p*
		1/3LD	2/3LD	3/3LD	1/3BFO	2/3BFO	3/3BFO	
IBW (g)	13.8 ± 0.01	13.8 ± 0.02	13.9 ± 0.04	14.0 ± 0.06	13.9 ± 0.03	13.9 ± 0.05	13.9 ± 0.01	0.097
FBW (g)	71.3 ± 2.20	73.0 ± 4.55	70.0 ± 1.41	65.9 ± 1.08	67.9 ± 2.24	67.3 ± 7.67	64.2 ± 3.88	0.720
WG (%)	397 ± 11.7	398 ± 38.4	394 ± 4.40	382 ± 13.5	360 ± 8.84	374 ± 47.5	371 ± 36.5	0.908
Survival (%)	95.8 ± 1.60	94.5 ± 4.01	97.8 ± 1.11	100 ± 0.00	94.5 ± 2.22	96.7 ± 3.34	100 ± 0.00	0.398
FI (%)	3.04 ± 0.13	2.66 ± 0.27	2.83 ± 0.20	2.85 ± 0.10	2.83 ± 0.28	2.98 ± 0.12	2.37 ± 0.05	0.343
FCR	1.02 ± 0.05	0.89 ± 0.09	0.97 ± 0.06	0.98 ± 0.03	0.98 ± 0.07	1.01 ± 0.05	0.84 ± 0.05	0.295
K (g/cm^3^)	3.48 ± 0.15	3.60 ± 0.19	3.52 ± 0.02	3.49 ± 0.10	3.46 ± 0.03	3.72 ± 0.12	3.41 ± 0.30	0.903
VSI (%)	15.7 ± 0.42	14.9 ± 0.29	15.2 ± 0.23	15.7 ± 0.21	16.2 ± 0.09	15.9 ± 0.56	14.9 ± 0.01	0.054
HSI (%)	10.3 ± 0.25	9.74 ± 0.22	10.1 ± 0.47	10.2 ± 0.21	10.6 ± 0.16	10.2 ± 0.78	9.68 ± 0.25	0.537

Evaluated by Tukey’s multiple test and one-way ANOVA.

**Table 5:** Proximate composition of whole body, muscle, and liver of experimental tiger puffer (% wet weight, mean ± standard error).

Parameters	MFO-C	Lard (LD)			Basa Fish Offal Oil (BFO)			*p*
		1/3LD	2/3LD	3/3LD	1/3BFO	2/3BFO	3/3BFO	
Whole fish								
Moisture	73.5 ± 0.81	74.9 ± 0.20	74.5 ± 0.40	74.4 ± 0.42	74.2 ± 0.50	74.9 ± 1.06	73.4 ± 1.75	0.702
Crude protein	16.8 ± 0.24	15.8 ± 0.36	16.3 ± 0.16	15.8 ± 0.52	16.9 ± 0.27	15.7 ± 0.34	17.0 ± 0.25	0.141
Crude lipid	5.34 ± 0.12	6.14 ± 0.18	5.64 ± 0.32	6.06 ± 0.24	6.03 ± 0.18	5.74 ± 0.28	5.35 ± 0.97	0.358
Ash	2.35 ± 0.03	2.50 ± 0.03	2.42 ± 0.06	2.39 ± 0.04	2.37 ± 0.09	2.37 ± 0.05	2.48 ± 0.07	0.438
Muscle								
Moisture	79.1 ± 0.41	79.2 ± 0.53	79.3 ± 0.30	78.9 ± 0.28	79.6 ± 0.20	79.8 ± 0.17	79.2 ± 0.20	0.615
Crude protein	18.1 ± 0.37	17.8 ± 0.79	18.0 ± 0.28	18.2 ± 0.41	17.9 ± 0.10	17.7 ± 0.29	18.3 ± 0.26	0.954
Crude lipid	2.37 ± 0.15 ^b^	2.46 ± 0.05 ^b^	2.10 ± 0.00 ^a^	2.38 ± 0.16 ^b^	2.09 ± 0.01 ^a^	2.39 ± 0.01 ^b^	2.47 ± 0.03 ^b^	0.032
Liver								
Moisture	26.8 ± 0.87	27.6 ± 1.42	29.5 ± 1.10	29.2 ± 1.24	29.4 ± 0.83	31.6 ± 1.84	31.6 ± 1.85	0.089
Crude lipid	41.5 ± 1.14	42.6 ± 0.42	38.2 ± 1.86	39.1 ± 1.58	43.2 ± 1.12	40.3 ± 1.45	38.1 ± 5.22	0.298

Data in the same row not sharing a superscript letter were significantly (*p <* 0.05) different, evaluated by Tukey’s multiple test and one-way ANOVA.

**Table 6:** Fatty acid compositions in the muscle of tiger puffer (%TFA, mean ± standard error).

Fatty Acid	MFO-C	Lard (LD)			Basa Fish Offal Oil (BFO)			*p*
		1/3LD	2/3LD	3/3LD	1/3BFO	2/3BFO	3/3BFO	
14:0	0.87 ± 0.18	0.49 ± 0.02	0.79 ± 0.21	0.54 ± 0.16	0.56 ± 0.03	0.64 ± 0.06	0.51 ± 0.05	0.502
15:0	2.58 ± 0.23 ^ab^	3.64 ± 0.71 ^ab^	3.14 ± 0.56 ^ab^	2.82 ± 0.37 ^ab^	1.90 ± 0.03 ^a^	3.17 ± 0.97 ^ab^	5.47 ± 0.60 ^b^	0.041
16:0	20.0 ± 0.72	19.2 ± 1.67	19.6 ± 0.72	18.9 ± 0.56	18.4 ± 0.19	19.2 ± 0.70	20.9 ± 0.73	0.560
17:0	1.31 ± 0.10	1.79 ± 0.35	1.68 ± 0.33	1.44 ± 0.18	1.01 ± 0.05	1.43 ± 0.40	2.27 ± 0.34	0.202
18:0	9.28 ± 0.40	10.1 ± 0.77	10.5 ± 0.40	10.2 ± 0.21	8.99 ± 0.07	9.73 ± 0.16	9.90 ± 0.33	0.151
SFA	34.3 ± 1.27	35.4 ± 3.34	36.0 ± 1.99	34.2 ± 1.10	31.2 ± 0.14	34.4 ± 2.07	39.2 ± 1.17	0.278
16:1n-7	1.09 ± 0.05	0.97 ± 0.11	1.09 ± 0.16	1.03 ± 0.17	0.91 ± 0.04	1.11 ± 0.30	0.71 ± 0.06	0.726
18:1n-9	11.2 ± 0.36 ^a^	13.5 ± 0.44 ^abc^	15.5 ± 0.76 ^cd^	16.7 ± 0.78 ^d^	12.3 ± 0.32 ^ab^	15.0 ± 0.64 ^bcd^	15.2 ± 0.70 ^bcd^	0.001
20:1n-9	0.23 ± 0.09	0.30 ± 0.15	0.20 ± 0.10	0.38 ± 0.07	0.37 ± 0.01	0.52 ± 0.15	0.13 ± 0.13	0.286
MUFA	13.4 ± 0.43 ^a^	15.9 ± 1.32 ^ab^	17.7 ± 0.89 ^ab^	18.9 ± 1.11 ^b^	14.2 ± 0.33 ^ab^	18.1 ± 1.22 ^ab^	17.0 ± 0.17 ^ab^	0.005
18:2n-6	7.80 ± 0.09 ^a^	10.3 ± 0.02 ^bc^	12.2 ± 0.05 ^de^	15.0 ± 0.16 ^f^	9.58 ± 0.18 ^b^	11.5 ± 0.62 ^cd^	13.3 ± 0.42 ^e^	0.001
20:2n-6	0.45 ± 0.04	0.43 ± 0.08	0.56 ± 0.10	0.65 ± 0.07	0.60 ± 0.02	0.62 ± 0.05	0.55 ± 0.06	0.196
20:4n-6	1.88 ± 0.11 ^ab^	1.60 ± 0.04 ^ab^	1.42 ± 0.12 ^ab^	1.22 ± 0.15 ^a^	1.97 ± 0.03 ^b^	1.69 ± 0.17 ^ab^	1.45 ± 0.13 ^ab^	0.018
n-6PUFA	10.3 ± 0.21 ^a^	12.4 ± 0.05 ^b^	14.2 ± 0.23 ^cd^	16.9 ± 0.33 ^e^	12.5 ± 0.20 ^bc^	14.3 ± 0.59 ^d^	15.7 ± 0.18 ^de^	0.001
18:3n-3	1.10 ± 0.03 ^d^	0.85 ± 0.06 ^bc^	0.65 ± 0.04 ^ab^	0.61 ± 0.07 ^ab^	0.96 ± 0.01 ^cd^	0.80 ± 0.06 ^abc^	0.52 ± 0.12 ^a^	0.001
20:5n-3	5.14 ± 0.28 ^b^	4.73 ± 0.18 ^ab^	3.96 ± 0.38 ^ab^	4.01 ± 0.18 ^ab^	5.21 ± 0.10 ^b^	4.13 ± 0.39 ^ab^	3.38 ± 0.26 ^a^	0.013
22:5n-3	3.07 ± 0.28	2.81 ± 0.02	2.88 ± 0.38	2.82 ± 0.06	3.28 ± 0.00	2.91 ± 0.17	2.53 ± 0.02	0.633
22:6n-3	21.8 ± 1.33 ^b^	19.0 ± 0.25 ^ab^	16.3 ± 1.81 ^ab^	15.8 ± 1.20 ^ab^	22.2 ± 0.02 ^b^	17.6 ± 1.91 ^ab^	13.4 ± 0.55 ^a^	0.013
n-3PUFA	31.2 ± 1.93 ^b^	27.4 ± 0.35 ^ab^	23.8 ± 2.59 ^ab^	23.3 ± 1.39 ^ab^	31.7 ± 0.06 ^b^	25.5 ± 2.42 ^ab^	19.9 ± 0.95 ^a^	0.015
n-3/n-6	3.03 ± 0.14 ^d^	2.21 ± 0.04 ^bc^	1.67 ± 0.15 ^ab^	1.37 ± 0.06 ^a^	2.53 ± 0.04 ^cd^	1.79 ± 0.20 ^ab^	1.26 ± 0.07 ^a^	0.001

In addition to the fatty acids listed in the table, the total fatty acids include 12:0, 13:0, 20:0, 21:0, 22:0, 14:1n-5, 15:1n-5, 17:1n-7, 22:1n-9, 18:3n-6, 20:3n-6, 22:2n-6, and 20:3n-3. Data in the same row not sharing a superscript letter were significantly (*p* < 0.05) different, evaluated by Tukey’s multiple test and one-way ANOVA.

**Table 7:** Fatty acid compositions in the liver of tiger puffer (%TFA, mean ± standard error).

Fatty Acid	MFO-C	Lard (LD)			Basa Fish Offal Oil (BFO)			*p*
		1/3LD	2/3LD	3/3LD	1/3BFO	2/3BFO	3/3BFO	
14:0	3.20 ± 0.13 ^c^	2.78 ± 0.13 ^abc^	2.35 ± 0.17 ^ab^	2.12 ± 0.06 ^a^	3.00 ± 0.10 ^bc^	2.68 ± 0.25 ^abc^	2.64 ± 0.03 ^abc^	0.001
16:0	24.6 ± 0.89	24.8 ± 0.59	23.3 ± 0.42	24.4 ± 0.39	24.8 ± 0.44	24.7 ± 0.67	26.0 ± 0.95	0.402
18:0	8.72 ± 0.24	10.1 ± 0.15	9.10 ± 0.32	9.51 ± 0.25	8.64 ± 0.30	8.72 ± 0.56	9.56 ± 0.28	0.053
SFA	37.7 ± 1.16	38.9 ± 0.66	35.6 ± 0.11	36.7 ± 0.60	37.5 ± 0.63	36.9 ± 0.51	38.8 ± 0.55	0.124
16:1n-7	7.28 ± 0.16 ^d^	5.91 ± 0.16 ^abc^	6.14 ± 0.34 ^bc^	5.39 ± 0.07 ^ab^	6.51 ± 0.21 ^cd^	5.55 ± 0.28 ^abc^	4.99 ± 0.19 ^a^	0.002
18:1n-9	21.5 ± 0.78 ^a^	24.7 ± 0.35 ^ab^	28.8 ± 1.75 ^bc^	31.7 ± 0.65 ^c^	24.8 ± 0.43 ^ab^	28.5 ± 0.25 ^bc^	32.1 ± 0.16 ^c^	0.001
20:1n-9	1.51 ± 0.08 ^c^	1.32 ± 0.06 ^abc^	1.38 ± 0.07 ^bc^	1.10 ± 0.02 ^ab^	1.36 ± 0.03 ^abc^	1.26 ± 0.03 ^abc^	1.07 ± 0.06 ^a^	0.003
MUFA	31.7 ± 0.81 ^a^	33.2 ± 0.32 ^ab^	37.6 ± 1.26 ^cd^	39.2 ± 0.58 ^d^	34.1 ± 0.47 ^abc^	36.6 ± 0.45 ^bcd^	39.1 ± 0.43 ^d^	0.001
18:2n-6	10.0 ± 0.16 ^a^	10.9 ± 0.06 ^ab^	11.5 ± 0.13 ^bc^	12.8 ± 0.34 ^c^	10.9 ± 0.31 ^ab^	11.6 ± 0.18 ^bc^	12.0 ± 0.88 ^bc^	0.001
20:2n-6	0.62 ± 0.06	0.66 ± 0.02	0.69 ± 0.02	0.74 ± 0.03	0.65 ± 0.02	0.69 ± 0.04	0.53 ± 0.11	0.174
20:4n-6	0.43 ± 0.03 ^b^	0.37 ± 0.04 ^ab^	0.34 ± 0.03 ^ab^	0.24 ± 0.01 ^a^	0.40 ± 0.03 ^b^	0.36 ± 0.01 ^ab^	0.24 ± 0.04 ^a^	0.001
n-6PUFA	11.3 ± 0.26 ^a^	12.1 ± 0.13 ^ab^	12.8 ± 0.10 ^bc^	13.8 ± 0.37 ^c^	12.3 ± 0.38 ^abc^	13.1 ± 0.24 ^bc^	13.1 ± 0.76 ^bc^	0.002
18:3n-3	2.48 ± 0.10 ^c^	1.97 ± 0.01 ^b^	1.72 ± 0.18 ^ab^	1.38 ± 0.05 ^a^	2.09 ± 0.06 ^bc^	1.72 ± 0.04 ^ab^	1.48 ± 0.17 ^a^	0.002
20:5n-3	4.12 ± 0.19 ^e^	3.47 ± 0.03 ^cde^	2.94 ± 0.24 ^cd^	2.16 ± 0.07 ^ab^	3.55 ± 0.17 ^de^	2.70 ± 0.08 ^bc^	1.82 ± 0.01 ^a^	0.001
22:5n-3	2.98 ± 0.22 ^c^	2.65 ± 0.10 ^bc^	2.49 ± 0.27 ^abc^	1.83 ± 0.05 ^ab^	2.55 ± 0.16 ^bc^	2.51 ± 0.05 ^abc^	1.69 ± 0.15 ^a^	0.003
22:6n-3	9.57 ± 0.61 ^d^	7.59 ± 0.27 ^cd^	6.63 ± 0.61 ^bc^	4.80 ± 0.19 ^ab^	7.66 ± 0.44 ^cd^	6.35 ± 0.30 ^bc^	3.99 ± 0.02 ^a^	0.003
n-3PUFA	19.3 ± 1.11 ^d^	15.8 ± 0.36 ^cd^	14.0 ± 1.28 ^bc^	10.3 ± 0.36 ^ab^	16.1 ± 0.72 ^cd^	13.5 ± 0.41 ^bc^	9.03 ± 0.22 ^a^	0.004
n-3/n-6	1.71 ± 0.06 ^d^	1.31 ± 0.02 ^c^	1.09 ± 0.10 ^bc^	0.74 ± 0.03 ^a^	1.30 ± 0.02 ^c^	1.02 ± 0.05 ^b^	0.69 ± 0.02 ^a^	0.001

In addition to the fatty acids listed in the table, the total fatty acids include 12:0, 13:0, 15:0, 17:0, 20:0, 21:0, 22:0, 14:1n-5, 15:1n-5, 17:1n-7, 22:1n-9, 18:3n-6, 20:3n-6, 22:2n-6, and 20:3n-3. Data in the same row not sharing a superscript letter were significantly (*p* < 0.05) different, evaluated by Tukey’s multiple test and one-way ANOVA.

**Table 8:** Biochemical parameters in serum and muscle of tiger puffer (mean ± standard error).

Parameters	MFO-C	Lard (LD)			Basa Fish Offal Oil (BFO)			*p*
		1/3LD	2/3LD	3/3LD	1/3BFO	2/3BFO	3/3BFO	
Muscle								
Protein carbonyl (nmol/mg)	0.87 ± 0.12 ^b^	0.38 ± 0.09 ^ab^	0.71 ± 0.17 ^ab^	0.20 ± 0.07 ^a^	0.65 ± 0.10 ^ab^	0.40 ± 0.20 ^ab^	0.44 ± 0.11 ^ab^	0.033
MDA (nmol/g)	0.52 ± 0.03 ^ab^	0.40 ± 0.05 ^ab^	0.33 ± 0.04 ^a^	0.33 ± 0.02 ^a^	0.55 ± 0.06 ^b^	0.32 ± 0.05 ^a^	0.38 ± 0.01 ^ab^	0.014
Serum								
TBA (umol/L)	3.87 ± 0.20	3.85 ± 0.19	4.30 ± 0.47	3.96 ± 0.45	3.19 ± 0.68	3.77 ± 0.03	3.77 ± 0.03	0.227
TC (mmol/L)	73.5 ± 9.41	71.2 ± 1.07	76.6 ± 1.51	68.0 ± 3.00	72.2 ± 0.28	78.2 ± 6.35	67.5 ± 6.02	0.375
TG (mmol/L)	5.12 ± 0.27	5.41 ± 0.12	4.94 ± 0.09	5.89 ± 0.43	5.45 ± 0.28	5.12 ± 0.53	5.15 ± 0.15	0.674
HDL-C (mmol/L)	0.90 ± 0.16	1.03 ± 0.18	0.93 ± 0.06	1.11 ± 0.18	0.87 ± 0.15	0.93 ± 0.08	0.77 ± 0.02	0.451
LDL-C (mmol/L)	5.87 ± 0.52	5.69 ± 0.46	6.17 ± 0.38	6.02 ± 0.46	6.75 ± 0.15	6.89 ± 0.04	5.01 ± 1.01	0.282
Protein carbonyl (nmol/mg)	5.47 ± 1.08	5.09 ± 0.55	4.74 ± 0.70	4.47 ± 0.40	4.80 ± 1.28	4.62 ± 0.44	3.16 ± 0.63	0.304
MDA (nmol/g)	9.86 ± 0.04 ^bc^	8.82 ± 0.58 ^bc^	6.52 ± 0.68 ^ab^	7.50 ± 0.14 ^abc^	9.00 ± 1.10 ^bc^	9.11 ± 0.81 ^bc^	5.77 ± 1.21 ^a^	0.022

Data in the same row not sharing a superscript letter were significantly (*p* < 0.05) different, evaluated by Tukey’s multiple test and one-way ANOVA.

**Table 9:** Relative mRNA expression levels of genes related to lipid metabolism in the liver of tiger puffer.

Gene	MFO-C	Lard (LD)			Basa Fish Offal Oil (BFO)			*p*
		1/3LD	2/3LD	3/3LD	1/3BFO	2/3BFO	3/3BFO	
Lipogenesis								
*fas*	1.00 ± 0.39	0.98 ± 0.38	0.55 ± 0.16	0.91 ± 0.33	1.02 ± 0.20	0.58 ± 0.05	0.51 ± 0.10	0.991
β-oxidation								
*cpt-1*	1.00 ± 0.26	0.76 ± 0.28	1.41 ± 0.42	1.28 ± 0.27	1.39 ± 0.53	0.62 ± 0.15	1.06 ± 0.20	0.672
*acox1*	1.00 ± 0.18	0.70 ± 0.22	0.76 ± 0.13	0.90 ± 0.33	1.27 ± 0.31	0.66 ± 0.07	0.56 ± 0.04	0.873
Biosynthesis of glycerides								
*gpat*	1.00 ± 0.28	1.49 ± 0.67	2.50 ± 1.42	1.19 ± 0.46	1.52 ± 0.28	0.83 ± 0.24	0.95 ± 0.04	0.722
*dgat1*	1.00 ± 0.47	0.54 ± 0.12	0.55 ± 0.32	0.55 ± 0.07	1.06 ± 0.51	0.69 ± 0.11	0.56 ± 0.04	0.764
*mgat2a*	1.00 ± 0.22	0.82 ± 0.29	0.80 ± 0.15	0.66 ± 0.05	0.62 ± 0.06	0.67 ± 0.04	0.58 ± 0.04	0.786
Hydrolysis of glycerides								
*atgl*	1.00 ± 0.57	0.81 ± 0.26	0.68 ± 0.37	2.29 ± 1.43	1.01 ± 0.39	0.53 ± 0.23	0.62 ± 0.41	0.863
*daglα*	1.00 ± 0.25	0.72 ± 0.14	1.08 ± 0.75	0.70 ± 0.12	1.26 ± 0.17	0.63 ± 0.21	0.65 ± 0.14	0.931
*hsl*	1.00 ± 0.37	1.02 ± 0.52	1.38 ± 0.68	3.78 ± 3.11	2.07 ± 0.93	0.54 ± 0.14	1.03 ± 0.47	0.600
*mgll*	1.00 ± 0.38	1.24 ± 0.73	1.58 ± 0.58	1.44 ± 0.29	3.20 ± 0.82	1.23 ± 0.61	0.78 ± 0.16	0.644
Lipid digestion								
*bsal*	1.00 ± 0.44	0.62 ± 0.57	1.50 ± 0.61	0.59 ± 0.40	0.72 ± 0.72	0.39 ± 0.19	0.49 ± 0.07	0.482
*lp*	1.00 ± 0.15	0.55 ± 0.10	1.18 ± 0.37	0.64 ± 0.16	0.98 ± 0.65	0.35 ± 0.15	0.49 ± 0.13	0.730
Lipid transport								
*lpl*	1.00 ± 0.25	0.53 ± 0.32	0.66 ± 0.16	1.17 ± 0.14	0.95 ± 0.21	0.69 ± 0.09	0.56 ± 0.14	0.791
*lipc*	1.00 ± 0.14	0.82 ± 0.38	0.70 ± 0.21	0.99 ± 0.40	0.57 ± 0.24	0.87 ± 0.14	0.98 ± 0.59	0.884
*fabp*	1.00 ± 0.29	7.97 ± 4.66	3.08 ± 1.30	1.56 ± 0.20	1.14 ± 0.89	0.59 ± 0.07	0.70 ± 0.23	0.313
*fatp1*	1.00 ± 0.09	1.06 ± 0.26	1.57 ± 0.35	1.11 ± 0.29	1.12 ± 0.37	1.08 ± 0.03	1.10 ± 0.32	0.733
*apoa1*	1.00 ± 0.32	0.59 ± 0.12	0.71 ± 0.12	0.83 ± 0.25	0.59 ± 0.08	0.97 ± 0.12	1.02 ± 0.29	0.703
*apoa4*	1.00 ± 0.32	0.43 ± 0.07	1.22 ± 0.39	0.94 ± 0.38	0.70 ± 0.24	0.66 ± 0.12	0.93 ± 0.01	0.252
*apob100*	1.00 ± 0.31	0.70 ± 0.10	0.70 ± 0.44	0.83 ± 0.57	0.82 ± 0.25	0.60 ± 0.20	0.63 ± 0.27	0.891
*apoe1*	1.00 ± 0.20	1.15 ± 0.13	1.59 ± 0.26	1.44 ± 0.28	0.54 ± 0.08	1.34 ± 0.22	1.76 ± 0.17	0.381
Lipid metabolism-related transcriptional factors								
*srebf1*	1.00 ± 0.26	1.42 ± 0.09	1.26 ± 0.38	1.59 ± 0.73	1.28 ± 0.18	0.74 ± 0.06	0.64 ± 0.09	0.970
*pparα1*	1.00 ± 0.12	1.39 ± 0.56	1.15 ± 0.57	1.03 ± 0.26	1.24 ± 0.39	0.49 ± 0.06	0.54 ± 0.00	0.843
*pparα2*	1.00 ± 0.32	1.52 ± 0.14	1.07 ± 0.15	1.79 ± 0.10	1.42 ± 0.20	0.58 ± 0.08	0.58 ± 0.03	0.781
*pparβ*	1.00 ± 0.14	0.71 ± 0.08	1.12 ± 0.37	1.24 ± 0.52	1.28 ± 0.31	0.65 ± 0.15	0.87 ± 0.08	0.831
*pparγ*	1.00 ± 1.36	0.79 ± 0.96	0.85 ± 0.14	1.11 ± 0.38	1.43 ± 0.63	1.02 ± 0.26	0.66 ± 0.30	0.954
*fxr*	1.00 ± 1.30	2.34 ± 0.30	1.19 ± 0.39	1.56 ± 0.41	1.87 ± 0.76	1.14 ± 0.29	1.22 ± 0.01	0.881
*lxrα*	1.00 ± 0.02	0.96 ± 0.09	1.95 ± 0.32	0.44 ± 0.12	0.99 ± 0.68	1.10 ± 0.64	0.08 ± 0.05	0.192
*hnf4α*	1.00 ± 1.49	0.81 ± 0.30	1.13 ± 0.25	0.83 ± 0.32	1.89 ± 0.55	0.96 ± 0.32	1.19 ± 0.07	0.570
*lrh-1*	1.00 ± 0.21	0.64 ± 0.29	0.93 ± 0.25	0.85 ± 0.14	1.35 ± 0.11	0.70 ± 0.20	0.87 ± 0.06	0.660
Cholesterol and bile acid biosynthesis								
*hmgcr*	1.00 ± 0.39	1.03 ± 0.41	0.66 ± 0.17	0.96 ± 0.30	0.82 ± 0.19	1.05 ± 0.12	0.69 ± 0.35	0.953
*cyp7a1*	1.00 ± 0.14 ^bc^	0.28 ± 0.07 ^a^	0.36 ± 0.10 ^ab^	0.53 ± 0.20 ^abc^	0.62 ± 0.06 ^abc^	0.91 ± 0.20 ^abc^	1.06 ± 0.11 ^c^	0.032

Data in the same row not sharing a superscript letter were significantly (*p* < 0.05) different, evaluated by Tukey’s multiple test and one-way ANOVA.

**Table 10:** Muscle texture indexes and water-holding capacity of tiger puffer (mean ± standard error).

Parameters	MFO-C	Lard (LD)			Basa Fish Offal Oil (BFO)			*p*
		1/3LD	2/3LD	3/3LD	1/3BFO	2/3BFO	3/3BFO	
Hardness (N)	2.22 ± 0.05	2.00 ± 0.11	2.07 ± 0.04	2.53 ± 0.42	1.61 ± 0.26	2.23 ± 0.45	2.23 ± 0.19	0.131
Adhesiveness (mJ)	0.03 ± 0.00	0.03 ± 0.00	0.41 ± 0.38	0.03 ± 0.00	0.03 ± 0.00	0.03 ± 0.01	0.03 ± 0.00	0.544
Cohesiveness (Ratio)	0.37 ± 0.01	0.37 ± 0.01	0.52 ± 0.13	0.47 ± 0.12	0.38 ± 0.00	0.42 ± 0.05	0.36 ± 0.01	0.693
Springiness (mm)	1.10 ± 0.06	1.00 ± 0.06	0.96 ± 0.16	1.16 ± 0.12	0.95 ± 0.09	1.00 ± 0.07	1.03 ± 0.03	0.720
Gumminess (N)	0.73 ± 0.06	0.77 ± 0.02	0.74 ± 0.04	0.78 ± 0.17	0.58 ± 0.07	0.77 ± 0.13	0.80 ± 0.01	0.470
Chewiness (mJ)	0.81 ± 0.12	0.77 ± 0.10	0.59 ± 0.25	1.00 ± 0.17	0.53 ± 0.10	0.79 ± 0.20	0.77 ± 0.05	0.461
Steaming water loss rate (%)	34.1 ± 1.85	35.1 ± 2.02	34.3 ± 1.58	32.9 ± 1.82	32.8 ± 3.17	34.2 ± 0.95	34.0 ± 1.30	0.984
Centrifugal water loss rate (%)	25.8 ± 0.67	25.4 ± 0.96	22.9 ± 1.30	24.4 ± 0.48	24.3 ± 1.79	23.3 ± 1.75	24.3 ± 0.32	0.670

Evaluated by Tukey’s multiple test and one-way ANOVA.

**Table 11:** Volatile flavor compounds identified in the muscle from the groups MFO-C, 3/3LD, and 3/3BFO.

Compounds	CAS Number	Formula	Relative Molecule Mass	Retention Index	Retention Time	Drift Time
Nonanal-M	C124196	C9H18O	142.2	1108.7	513.634	1.4737
Nonanal-D	C124196	C9H18O	142.2	1108.7	513.634	1.94818
Octanal-M	C124130	C8H16O	128.2	1007.8	362.504	1.4013
Octanal-D	C124130	C8H16O	128.2	1007.4	362.024	1.82648
1-Heptanol	C111706	C7H16O	116.2	989.4	341.874	1.39976
oct-1-en-3-ol	C3391864	C8H16O	128.2	983.8	336.596	1.16252
Benzaldehyde-M	C100527	C7H6O	106.1	962.1	316.925	1.15482
Cyclohexanone	C108941	C6H10O	98.1	898.3	265.589	1.15174
Heptanal-M	C111717	C7H14O	114.2	899.5	266.458	1.33141
Heptanal-D	C111717	C7H14O	114.2	899.7	266.636	1.70068
Hexanal-M	C66251	C6H12O	100.2	796.2	208.575	1.26007
Hexanal-D	C66251	C6H12O	100.2	794.6	207.758	1.56723
(E)-2-hexenal-M	C6728263	C6H10O	98.1	847.1	234.981	1.18272
pentan-1-ol-M	C71410	C5H12O	88.1	763.1	192.785	1.25439
Methyl isobutyl ketone-M	C108101	C6H12O	100.2	735.8	180.535	1.1793
Methyl isobutyl ketone-D	C108101	C6H12O	100.2	733.9	179.719	1.48304
Benzaldehyde-D	C100527	C7H6O	106.1	960.8	315.857	1.47535
n-Hexanol	C111273	C6H14O	102.2	867.6	246.515	1.32608
(E)-2-hexenal-D	C6728263	C6H10O	98.1	847.3	235.064	1.51856
2-Heptanone	C110430	C7H14O	114.2	887.6	258.343	1.25948
(E)-2-pentenal-M	C1576870	C5H8O	84.1	751.7	187.586	1.10976
Methyl isobutyl ketone-D	C108101	C6H12O	100.2	733.9	179.719	1.48304
Benzaldehyde-D	C100527	C7H6O	106.1	960.8	315.857	1.47535
n-Hexanol	C111273	C6H14O	102.2	867.6	246.515	1.32608
(E)-2-hexenal-D	C6728263	C6H10O	98.1	847.3	235.064	1.51856
2-Heptanone	C110430	C7H14O	114.2	887.6	258.343	1.25948
(E)-2-pentenal-M	C1576870	C5H8O	84.1	751.7	187.586	1.10976
(E)-2-pentenal-D	C1576870	C5H8O	84.1	748.9	186.326	1.36418
pentan-1-ol-D	C71410	C5H12O	88.1	761	191.785	1.51165
(E)-hept-2-enal	C18829555	C7H12O	112.2	947.4	304.325	1.26398
(E)-3-penten-2-one	C3102338	C5H8O	84.1	738.9	181.917	1.09174
Propanoic acid	C79094	C3H6O2	74.1	715.2	171.838	1.10976
Pentanal-D	C110623	C5H10O	86.1	701.5	166.286	1.42736
2,3-pentanedione	C600146	C5H8O2	100.1	690.3	161.894	1.22403
Pentanal-M	C110623	C5H10O	86.1	699	165.31	1.18635
Ethyl Acetate	C141786	C4H8O2	88.1	612.7	140.585	1.33708
2-Butanone	C78933	C4H8O	72.1	579.6	132.452	1.24837
Butanal	C123728	C4H8O	72.1	538.7	123.018	1.28998
3-Pentanone	C96220	C5H10O	86.1	697	164.496	1.3622
2-Pentanone	C107879	C5H10O	86.1	686.4	160.593	1.38104
2-Methylbutanal	C96173	C5H10O	86.1	661.7	153.598	1.16123
3-Methylbutanal	C590863	C5H10O	86.1	652.8	151.158	1.17379
2-Hexanone	C591786	C6H12O	100.2	778	199.794	1.18871
acetone	C67641	C3H6O	58.1	491.3	112.933	1.11805
2,3-butanedione	C431038	C4H6O2	86.1	578.3	132.127	1.17065
methyl acetate	C79209	C3H6O2	74.1	529.1	120.903	1.20048
ethyl propanoate	C105373	C5H10O2	102.1	710.3	169.864	1.15181
Methylpyrazine	C109080	C5H6N2	94.1	798.8	209.841	1.07943
2,3-Hexanedione	C3848246	C6H10O2	114.1	775	198.329	1.27764

## Data Availability

Raw data supporting the conclusions of this manuscript will be made available by the authors, without undue reservation, to any qualified researcher.

## Abbreviations

Non-gene name	
MFO	marine fish oil
LC-PUFA	long-chain polyunsaturated fatty acids
EPA	eicosapentaenoic acid
DHA	docosahexaenoic acid
SFA	saturated fatty acid
MUFA	monounsaturated fatty acid
PUFA	polyunsaturated fatty acids
LD	lard
BFO	basa fish offal oil
HSI	hepatosomatic index
VSI	visecrosomatic index
K	condition factor
AOAC	association of official analytical chemists
TFA	total fatty acid
MDA	malondialdehyde
TBA	total bile acid
TC	total cholesterol
TG	triacylglycerol
HDL-C	high-density lipoprotein cholesterol
LDL-C	low-density lipoprotein cholesterol
RT-qPCR	real-time quantitative polymerase chain reaction
WG	weight gain
FBW	final body weight
IBW	initial body weight
FI	feed intake
FCR	feed conversion ratio
Gene name	
*fas*	fatty acid synthase
*cpt-1*	carnitine O-palmitoyltransferase-1
*acox1*	acyl-CoA oxidase 1, palmitoyl
*gpat*	glycerol-3-phosphate acyltransferase
*dgat1*	diacylglycerol O-acyltransferase 1
*mgat2a*	2-acylglycerol O-acyltransferase 2-A-like (LOC101069338)
*atgl*	adipose triglyceride lipase (patatin like phospholipase domain containing 2 (*pnpla2*))
*daglα*	diacylglycerol lipase, alpha
*hsl*	hormone-sensitive lipase
*mgll*	monoglyceride lipase
*bsal*	bile acid activated lipase
*lp*	inactive pancreatic lipase-related protein 1-like (LOC101064949)
*lpl*	lipoprotein lipase
*lipc*	lipase, hepatic
*fabp*	fatty acid binding protein
*fatp*	fatty acid transport protein (solute carrier family 27 member 1 (*slc27a1*))
*apo*	apolipoprotein
*srebf1*	sterol regulatory element binding transcription factor 1
*ppar*	peroxisome proliferators-activated receptor
*fxr*	farnesoid X receptor (nuclear receptor subfamily 1, group H, member 4, *nr1h4*)
*lxrα*	liver X receptor alpha (nuclear receptor subfamily 1, group H, member 3, *nr1h3*)
*hnf4α*	hepatocyte nuclear factor 4, alpha
*lrh-1*	liver receptor homolog-1 (nuclear receptor subfamily 5, group A, member 2, *nr5a2*)
*hmgcr*	3-hydroxy-3-methylglutaryl-CoA reductase
*cyp7a1*	cholesterol 7-alpha-hydroxylase (cytochrome P450 7A1)
